# Revisiting the dynamic interactions between economic growth and environmental pollution in Italy: evidence from a gradient descent algorithm

**DOI:** 10.1007/s11356-021-14264-z

**Published:** 2021-05-18

**Authors:** Marco Mele, Cosimo Magazzino, Nicolas Schneider, Floriana Nicolai

**Affiliations:** 1 Department of Political Sciences, Roma Tre University, Rome, Italy; 2 Department of Economics, Paris-1 Pantheon-Sorbonne University, Paris, France; 3 Rome, Italy

**Keywords:** CO_2_ emissions, Economic growth, Italy, Machine learning, Environmental policy, B22, C32, N55, Q43

## Abstract

Although the literature on the relationship between economic growth and CO_2_ emissions is extensive, the use of machine learning (ML) tools remains seminal. In this paper, we assess this nexus for Italy using innovative algorithms, with yearly data for the 1960–2017 period. We develop three distinct models: the batch gradient descent (BGD), the stochastic gradient descent (SGD), and the multilayer perceptron (MLP). Despite the phase of low Italian economic growth, results reveal that CO_2_ emissions increased in the predicting model. Compared to the observed statistical data, the algorithm shows a correlation between low growth and higher CO_2_ increase, which contradicts the main strand of literature. Based on this outcome, adequate policy recommendations are provided.

**Supplementary Information:**

The online version contains supplementary material available at 10.1007/s11356-021-14264-z.

## Introduction

Global warming has become an increasing threat worldwide. In the near future, sea level rises and weather shocks are expected to be more frequent, making agricultural farmers and coastal populations more vulnerable. This is corroborated by some recent evidence: due to elevated greenhouse gas (GHG) emissions trends, 2015–2017 were the three warmest years of the pre-modern history (Liu et al. 2019). Heavily promoted by the Intergovernmental Panel on Climate Change’s reports (IPCC 2007, 2011), the adoption of low-carbon strategies takes center stage in the current environmental debate. In the OECD, power-based renewable resources are in constant expansion. However, far from being limited to high-income countries, key renewable energy leaders with encouraging technological features (China and India notably) are also emerging in Asia (Magazzino et al. 2021c). While significant room for improvements remains, a global carbon mitigation dynamic has started. In this process to turn societies toward a sustainable path, the role of Italy should be strengthened.

Stuck in an economic crisis, the Italian government relied on competition leverages to restore productive returns and reach economic targets (Bento and Moutinho 2016). Unfortunately, a collateral cost of this growth strategy is the environment. Put aside for the sake of economic performance, the energy transition has not yet been completely framed. Hence, Italy has been trapped in a growth-environment dilemma but seeks to reconcile these objectives in the long run. Key local benefits can be drawn from carbon dioxide (CO_2_) mitigation. It has been demonstrated that elevated levels of polluting particles may adversely affect productivity (Agrawal et al. 2003), cognitive performance (Ebenstein et al. 2016), crime (Herrnstadt et al. 2020), and health (Ebenstein et al. 2017). Although they share central channels with society, the costs associated with air pollution were long ignored by environmental regulators in the past. Nowadays, they remain insufficiently internalized within energy planning, notably in Italy.

At the aggregate level, CO_2_ emissions from fuel combustion increased from 289,481 to 302,775 thousand tons over the period 1971–2019. Looking in closer scrutiny, we notice that aggregate emissions have experienced a decreasing path since 1990, although their absolute levels remain critically high. As explained in Bento and Moutinho (2016, b), Italy has a strong industrial base that triggers its energy needs. Since 1998, a constant surplus of exports has been recorded and accompanied by a booming segment of services. Unsurprisingly, the difficulty of the most intensive sectors to control their demand for energy over decades slowed down the transition toward a decarbonized economy. However, it does not mean that energy efficiency technologies and fuel substitution strategies are absent in the key industries. Between 1990 and 2019, CO_2_ emissions from the power and heating sector recorded a decrease from 123,981 to 95,878 thousand tons. In the meantime, electricity generation from renewables (excluding hydroelectric) rose from 1.5 to 22.5%. Being long considered at the core of pollution, the electricity sector stands now at the heart of the environmental solution. Similarly, the level of carbon released from manufacturing industries and construction decreased from 74,026 to 32,256 thousand tons over the same period. Although the economic crisis might have played a significant role in this dynamic, combined policy measures may also explain these declining pollution trends. For instance, the National Energy Efficiency Action Plan 2014 projected to save 20 Mtoe/year of primary energy and 15.5 Mtoe/year of final energy by the end of the past decade (IEEAP, 2014). In addition, the new energy-saving decree (Italian Ministerial Decree of 12/21/2017) imposes a cumulative end-use energy savings objective of 25 Mtoe/year (Malinauskaite et al., 2019). Ultimately, the 2017 Italian National Energy Strategy will invest EUR110 billion to reduce energy use of around 10 Mtoe/year by 2030 (ENEA, 2018). Along with carbon reductions, per capita income presents an upward trend: from $32,155 in 2014, it reached $34, 318 in 2018 (Acar and Lindmark 2017). While this path is propitious, further commitments must follow to achieve an absolute delinking between environmental degradation and economic performance. As a matter of fact, 60.2% of the power produced is still sourced from fossil fuels. This is of high concern in a country that is ranked as the 4^th^ largest energy consumer in Europe (Malinauskaite et al., 2019).

Accordingly, there is a point in investigating here the dynamic interactions between economic activity and CO_2_ emissions in Italy. We hereby ask under which conditions the growth of income may operate as a sustainability enabler and to what extent revising downwards the economic targets (because of an economic recession) may also hinder environmental objectives. Looking at the literature, key gaps can be highlighted based on which novelty aspects can be drawn. Above all, the relationship between GDP and CO_2_ emissions has been abundantly studied through the lens of the environmental Kuznets curve (EKC) (Stern and Common, 2001; Acaravci and Ozturk, 2010; Bilgili et al., 2016). Nonetheless, econometric methodologies, data, and results differ and sometimes conflict. Second, while a range of papers included Italian data within larger panel assessments, few studies have been conducted using a single-country approach (Annicchiarico et al., 2014; Bento and Moutinho, 2016; Stamatiou and Dritsakis, 2019). Here, we claim that this has non-negligible advantages. On the one hand, the generalization of panel findings to each member is questionable and may be inconsistent. Economies present structural divergences and generally design their policies according to their own characteristics. For this reason, examining a unique case study is more likely to provide reliable country-specific insights for policy purpose. Third and finally, to the best of our knowledge, no study inspected the GDP-CO_2_ nexus in Italy using a machine learning (ML) approach. This is surprising since techniques derived from artificial intelligence (AI) have already brought valuable evidence on neighboring topics (Magazzino et al., 2020a, 2020b, 2021a, 2021b, 2021c, 2021d). Undoubtedly, they shed light on another critical lack in the literature.

Therefore, this paper strives to fill the above-mentioned gaps in a single manner and presents the first empirical assessment of the relationship between economic growth and CO_2_ emissions in Italy using an innovative ML approach. To do so, we develop three distinct models, namely, the batch gradient descent (BGD), the stochastic gradient descent (SGD), and the multilayer perceptron (MLP). While the first two aim at optimizing the prediction of carbon dioxide emissions by taking GDP product as an input variable and CO_2_ emissions as the output variable, the latter one seeks to check the robustness of the predicted values and the strength of the correlation link. Data span the largest available period (1960–2016). Our original results are thought to open a new research direction and draw relevant policy insights for environmental regulators.

Besides the “Introduction” section, the remainder of the study proceeds as follows. The “Literature review” section provides a state-of-the-art review of the topic. The “Data and methodological framework” section describes the data and set up the theoretical ML framework. In the “Results and discussion” section 4, results of the BGD and SGD algorithm are displayed. The “ANN and MLP models” section presents the ANN and MLP models, while in the “MLP algorithms” section 6, empirical results are discussed. Finally, in “Concluding remarks and policy implications” section, concluding remarks and policy insights are delivered.

## Literature review

In this survey, we first present the panel EKC studies on the relationship between economic growth and environmental pollution (“Economic growth-carbon emissions nexus: multi-country studies” section). Then, we overview the few examinations that have been conducted on the single Italian case (“The relationship between economic activity and environmental pollution in Italy” section).

### Economic growth-carbon emissions nexus: multi-country studies

The relationship between economic growth and CO_2_ emissions has been widely used through the EKC by reference to the seminal Kuznets (1967) contribution. Traced back to Grossman et al. (1991), this hypothesis supports that environmental pollution first rises with economic development. However, after a certain turning point, the trend reverses, and the basic conflict among these two entities becomes solved. When confirmed, the relationship between economic and environmental indicators exhibits a major non-linearity, taking the form of an inverted U-shaped curve. In this case only, it is said that a delinking relationship emerges among these indicators. Often, the authors of these studies included energy consumption data to test whether the validation of the EKC is sensitive to the energy channel.

The literature is rich in empirical assessments that support this hypothesis based on data collected from large groups of countries (OECD, EU, G-7, high-income countries, developing economies, or mixed samples). Studies applied a wide range of different econometric procedures. Upon the most relevant studies, one finds Grossman et al. (1991) for 42 countries, Shafik and Bandyopadhyay (1992) for 149 countries, Panayotou (1993) for 68 countries, Stern and Common (2001) for 73 countries, Apergis and Payne (2009) for 6 central American countries, Leitao (2010) for 94 economies, Jaunky (2011) for 36 high-income countries, Iwata et al. (2012) for 11 OECD countries, Bilgili et al. (2016) for 17 OECD countries, Kais and Sami (2016) for 58 countries, Zaman and Abd-el Moemen (2017) for 90 economies, and Alshubiri and Elheddad (2019) for 32 OECD countries. More recently, this theory found empirical support in Adeel-Farooq et al. (2020) for 6 ASEAN countries, Dogan and Inglesi-Lotz (2020) for 7 EU countries, and Leal and Marques (2020) for 20 OECD countries. Inversely, the EKC hypothesis has been rejected in Acaravci and Ozturk (2010) for 19 EU countries, Narayan and Narayan (2010) for 43 developing countries, Antonakakis et al. (2017) for 106 countries, Cai et al. (2018) for G-7 countries, and Pata and Aydin (2020) for 6 hydropower consuming countries. Finally, mixed EKC outputs have been reported in Lee et al. (2010) since the authors found evidence of the inverted U-shaped relationship in America and Europe only. All in all, Isik et al. (2021) confirmed the EKC hypothesis for 4 out of 8 OECD countries.

Table 1 summarizes the main information (author(s), sample of countries, period of time, econometric methodology, energy data, main EKC finding) of this literature. Also, extensive surveys can be found in Saboori et al. (2012).

**Table 1:** Summary of the most relevant multi-country EKC studies on the GDP-environmental pollution nexus

Author(s)	Sample	Period	Methodology	Energy data	EKC
Grossman et al. (1991)	42 countries	1977–1988	FE	-	Yes
Shafik and Bandyopadhyay (1992)	149 countries	1960–1990	FE	-	Yes
Panayotou (1993)	68 countries	1988	OLS	T	Yes
Stern and Common (2001)	73 countries	1960–1990	FE, RE	-	Yes
Apergis and Payne (2009)	6 central American countries	1971–2004	VECM	T	Yes
Acaravci and Ozturk (2010)	19 EU countries	1960–2005	ARDL	T	No
Leitao (2010)	94 countries	1981–2000	FE, RE	T	Yes
Lee et al. (2010)	97 countries	1980–2001	GMM	-	Mixed
Narayan and Narayan (2010)	43 developing countries	1980–2004	Panel cointegration	-	No
Jaunky (2011)	36 high-income countries	1980–2005	GMM and VECM	-	Yes
Iwata et al. (2012)	11 OECD countries	1960–2003	ARDL	NE	Yes
Bilgili et al. (2016)	17 OECD countries	1977–2010	FMOLS, DOLS	R	Yes
Kais and Sami (2016)	58 countries	1990–2012	GMM	T	Yes
Antonakakis et al. (2017)	106 countries	1971–2011	VAR, IRF	T	No
Zaman and Abd-el Moemen (2017)	90 countries	1975–2015	GMM	T	Yes
Cai et al. (2018)	G-7 countries	1965–2015	ARDL	R	No
Alshubiri and Elheddad (2019)	32 OECD countries	1990–2015	GMM, FE	-	Yes
Adeel-Farooq et al. (2020)	6 ASEAN countries	1985–2012	MG, PMG	-	Yes
Dogan and Inglesi-Lotz (2020)	7 EU countries	1980–2014	PPC, FMOLS	T	Yes
Leal and Marques (2020)	20 OECD countries	1990–2016	ARDL	F/R	Yes
Pata and Aydin (2020)	6 hydropower consuming countries	1965–2016	ARDL	R	No
Isik et al. (2021)	8 OECD countries	1962–2015	CCEMG	T	Mixed

Source: our elaborations

T, F, R, and NE refer to total energy consumption, fossil fuel energy consumption, renewable energy consumption, and nuclear energy consumption, respectively. - indicates that no energy consumption data were included in the estimation model. “Yes” indicates that the EKC hypothesis is supported, while “No” designates its empirical rejection. *ARDL*: autoregressive distributed lag, *CCEMG* common correlated effects mean group, *DOLS* dynamic ordinary least squares, *FE* fixed effects, *FMOLS* fully modified ordinary least squares, *GMM* generalized method of moments, *IRF* impulse response function, *MG* mean group, *OLS* ordinary least squares, *PPC* Pedroni panel cointegration, *RE* random effects, *VAR* vector autoregressive, *VECM* vector error correction model

### The relationship between economic activity and environmental pollution in Italy

A few studies assessed the GDP-pollution nexus for the single Italian case. In a seminal work, Andreoni and Galmarini (2012) applied a decomposition analysis (DA) on Italy and showed that, over the 1998–2006 period, this economy did not achieve a significant decoupling between energy use, economic activity, and carbon emissions. In Mazzanti et al. (2008), the authors provided evidence supporting the EKC hypothesis for CO_2_, CH_4_, and CO. Germani et al. (2014) analyzed the relationship between income, demographic characteristics, and environmental pollution in Italy and validated the existence of the EKC: carbon emissions increase with income up to a turning point where the relation reverts. Annicchiarico et al. (2014) explored the relationship between economic growth and CO_2_ emissions in Italy using data spanning the 1861–2011 period. Findings derived from the MS-VAR model highlighted that GDP growth and carbon dioxide emissions are strongly interrelated. This contradicts with Bento and Moutinho, 2016, b) who showed that economic growth leads to less pollution over time, which is congruent with the EKC hypothesis. Using a VAR model and the Toda-Yamamoto causality test, Magazzino (2016) found a bidirectional link between energy consumption and CO_2_ emissions, as well as between economic growth and CO_2_ emissions for Italy. Lastly, Stamatiou and Dritsakis (2019) inspected the nexus between energy consumption, economic growth, and CO_2_ emissions in Italy from 1960 to 2011. VECM results revealed the existence of a one-way causality from GDP to CO_2_ emissions. This is in line with the conclusion drawn previously in Stamatiou and Dritsakis (2017). Table 2 outlines the main information of this literature.

**Table 2:** Summary of previous assessments of the GDP-CO_2_ emissions nexus in Italy

Author(s)	Country	Period	Methodology	Finding
Mazzanti et al. (2008)	Italy	1990–2001	OLS	Y→↓ CO_2_ (EKC)
Andreoni and Galmarini (2012)	Italy	1998–2006	DA	E→ CO_2_ and Y→ CO_2_
Annicchiarico et al. (2014)	Italy	1861–2011	MS-VAR	Y→ CO_2_
Germani et al. (2014)	Italy	2001–2005	OLS	Y→↓ CO_2_ (EKC)
Bento and Moutinho (2016, b)	Italy	1960–2011	ARDL, GC	(RE, Y) →↓ CO_2_ (EKC)
Magazzino (2016)	Italy	1970–2006	VAR, TY	E↔ CO_2_ and Y↔ CO_2_
Stamatiou and Dritsakis (2017)	Italy	1960–2011	VECM	Y→E and Y→ CO_2_
Stamatiou and Dritsakis (2019)	Italy	1960–2011	VECM, IRF	Y→ CO_2_

Source: our elaborations

Y, CO_2_, E, and RE refer to economic growth, carbon dioxide emissions, energy consumption, and renewable energy consumption, respectively. A→B indicates the existence of a unidirectional causality from A to B. A↔B indicates that a bidirectional causality between A and B is supported. A→↓B indicates that as A increases, B decreases. *AMS-VAR* Markov-switching vector autoregressive, *ARDL* autoregressive distributed lag, *DA* decomposition analysis, *GC* Granger causality, *IRF* impulse response function, *OLS* ordinary least squares, *VECM* vector error correction model, *TY* Toda-Yamamoto. (EKC) indicates that the EKC is confirmed

Using a slightly different approach, other non-carbon-related investigations have been performed the single Italian case and brought a specific focus on the linkages operating between energy, economy, and finance. Upon them, we can highlight Magazzino (2012), Magazzino and Giolli (2014), and Magazzino (2015) for the total energy-GDP relationship; Magazzino (2017) and Brady and Magazzino (2018) for the renewable energy consumption-income nexus; and Magazzino (2018) for the energy consumption-financial development nexus in Italy. Overall, recent assessments of the energy-GDP, energy-CO_2_, waste-GDP, waste-CO_2_ nexuses using ML tools and for various case studies can be found in Mele and Magazzino (2020), Magazzino et al. (2020a, 2020b, 2021a, 2021b, 2021c, 2021d), and Mele et al. (2021). Nonetheless, one must admit that such an innovative methodology has not been applied to the relationship between economic growth and CO_2_ emissions in Italy. Accordingly, this paper seeks to extend this econometric-based literature by bringing novel ML evidence on this nexus. This has the advantage to overcome the standard statistical issues often underlined in past time-series analyses while providing reliable and consistent evidence for Italy. Findings are thought to be of interest for policymakers.

## Data and methodological framework

Unlike almost all the studies conducted on this research subject, which use econometric methodologies, we apply advanced ML techniques. An experiment in ML can learn significant parameters from a set of known information to generate new knowledge, equipped with meaning and used to perform a particular task. There are many algorithms used to make estimates in ML. Each can be adapted, through new writing of the commands, to the estimation needs to be made. In this regard, we have changed and implemented two approaches. The first uses the analysis of the DG and the second a multilayer perceptron algorithm. Below, we illustrate the data and sources used of the first model, with the main commands used for the analysis. Next, we introduce the second algorithm. The study uses yearly data on Italy from 1960 to 2017. The analysis uses CO_2_ emissions and GDP growth as dependent and independent variables, respectively. The GDP data is expressed in millions of euros. The data source is the World Development Indicator (WDI)^1^.

The GD is an iterative optimization algorithm to find the minimum of a function. Here that function is our loss function of linear regression. The GD optimization algorithm is the main component in training the predictive model. There are two metrics for GD optimizer for its performance (1) generalization (model’s prediction for out of sample data) and (2) time taken for converging within the tolerance limit. This study used two models: BGD and SGD.

### BGD: construction of the model

For implementing the BGD, all the training data is taken into consideration to take a single step. The research took the average of all the training samples’ gradients and then used that mean gradient to update the parameters—that is, one step of gradient descent in one epoch. Consequently, the study expected the graph of cost against epochs to be quite smooth because the gradients of training data for a single step were being averaged.

Linear regression cost function:

$$ J\left(\varnothing \right)=\frac{1}{2m}{\sum}_{i=1}^m{\left({y}^{\ast i}-{y}^i\right)}^2 $$

where *m* = training data → $\frac{\partial }{\partial {\varnothing}_j}J\left(\varnothing \right)=\frac{1}{m}{\sum}_{i=1}^m\left({y}^{\ast i}-{y}^i\right){x}_j^i$ and BGD is:

$$ {\varnothing}_j={\varnothing}_j-\alpha \frac{\partial }{\partial {\varnothing}_j}J\left(\varnothing \right) $$

where $$ \frac{\partial }{\partial {\varnothing}_j}J\left(\varnothing \right) $$ = $$ {\sum}_{i=1}^m\left({y}^{\ast i}-{y}^i\right){x}_j^i $$

In this context, the Python codes are as follows:

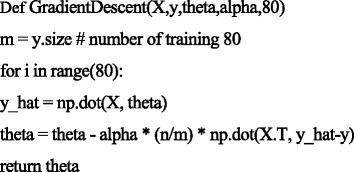



### SGD: construction of the model

In SGD, the study has considered just one sample at a time to take a single step. The cost fluctuates over the training samples and does not necessarily decrease. However, in the end, the cost starts to decrease with fluctuations. SGD converges faster when the dataset is larger.

Linear regression cost function:

$$ J\left(\varnothing \right)=\frac{1}{2m}{\sum}_{i=1}^m{\left({y}^{\ast i}-{y}^i\right)}^2 $$

where *m* = training data → *i* in range (*m*) →${\varnothing}_j={\varnothing}_j-\alpha \left({y}^{\ast i}-{y}^i\right){x}_j^i$

In this context, the Python codes area as follows:

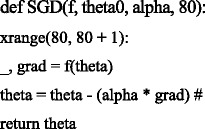



### Predictive model building procedure

Step 1 In this step, the study collected the data for the model, as the process also involves the ingestion of the data into python software for processing and analysis.
Step 2 The research separates the data into two: the input variable (GDP growth) and the output variable (CO_2_ emissions).
Step 3 In the third step, an exploratory analysis of the input and output variables have been conducted to plot scatter to assess the relationship between the two variables. Further, both independent and dependent variables are split at a ratio of 80:20 for training and testing of the model.
Step 4 In this step, training and testing losses were plotted against epochs at around epoch number 60 training. At this iteration number, validation losses are not decreasing further as the epochs are increased so that the ideal epoch would be 60 in this case.
Step 5 The best performing model between SGD and BGD was selected through observing the prediction results based on the root mean squared error (RMSE).

## Results and discussion

In Table 3, the mean presents a positive value for CO_2_ and GDP; 10-Trim values are near the mean; and the IQR shows the absence of outliers.

**Table 3:** Descriptive statistics

Statistics	CO_2_	GDP
Mean	6.33	27,097.23
Median	6.65	29,294.12
Std. dev.	1.52	8539.96
Skewness	−1.11	−0.41601
Kurtosis	0.54	−1.1936
10-Trim	6.39	27159.14
IQR	1.81	1.73

Our elaborations in Stata

**Fig. 1:**
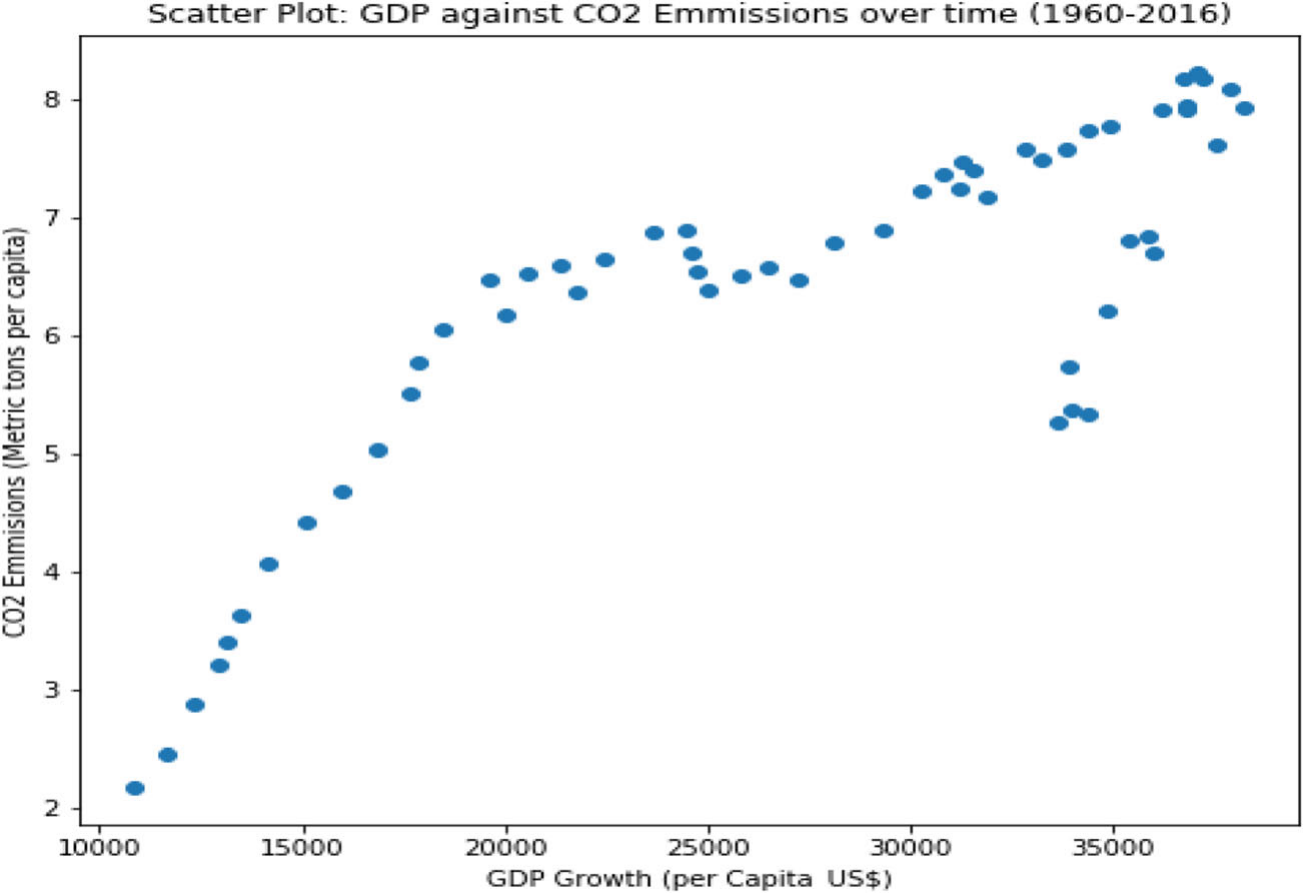
Scatter plot GDP against CO_2_ emission (Italy, 1960–2016). Source: our elaborations in Python 3.10

Figure 1 shows a non-linear relationship between the two variables suggesting the need to use more advanced machine learning algorithms that can learn non-linear and more sophisticated mathematical mapping.

**Fig. 2:**
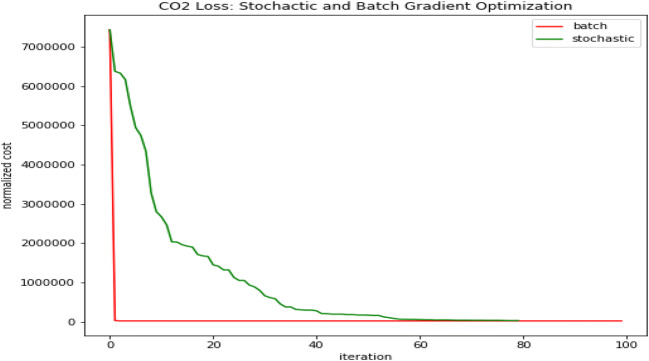
SGD and BGD optimization. Source: our elaborations in Python 3.10

The prediction loss graph indicates that the training loss is not decreasing beyond the 60th iteration for both the BGD and SGD optimizers. Therefore, the study took 60 as the ideal epoch for further prediction of the model (Fig. 2).

The parameters of BGD and SGD algorithms for predicting the output variable (CO_2_) are presented in Table 4. In both models, 80% of the data are used for model training and 20% used for model testing. The study compares the prediction strengths of the models in terms of root mean squared error; the lower the RMSE, the better the prediction capacity of the model. From the results, the BGD is having a higher predictive capacity than the SGD algorithm.

**Table 4:** BGD and SGD parameters

	BGD CO_2_ optimizer	SGD CO_2_ optimizer
RMSE	163.84	197.68
*T*_0_	25.73	19.09
*T*_1_	14.60	16.49

Source: our elaborations in Python

**Fig. 3:**
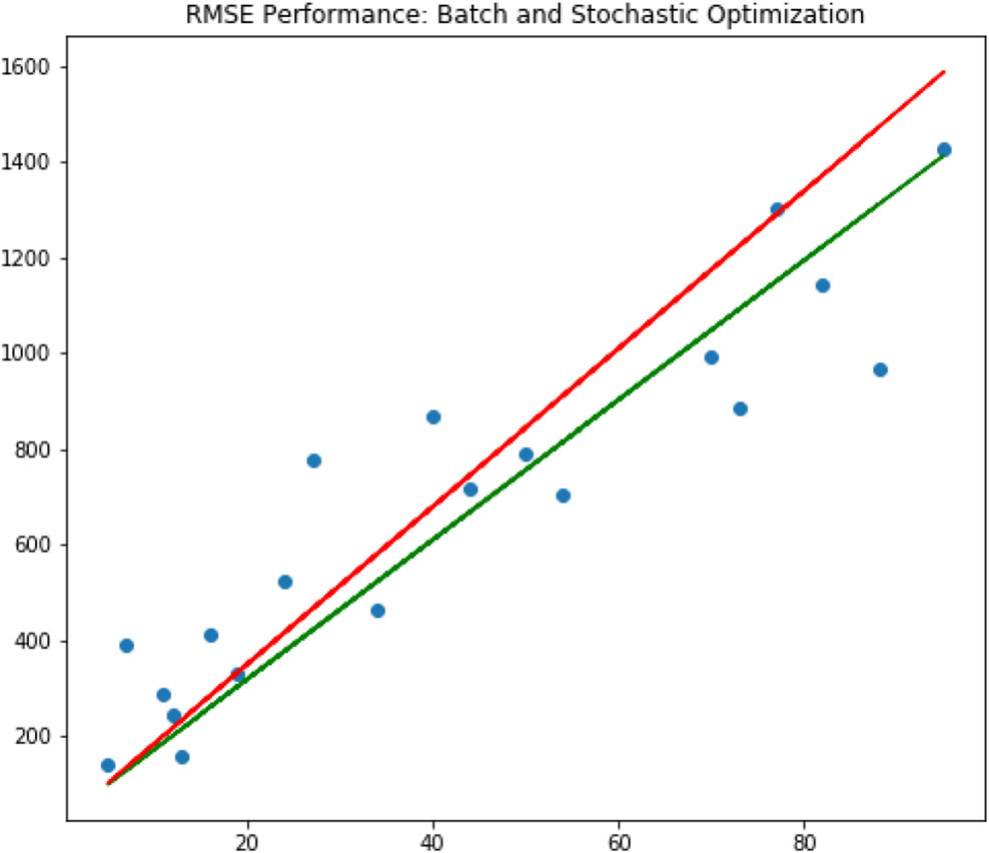
RMSE performance for batch and stochastic optimization. Source: our elaborations in Python 3.10

In this first study, linear regression with a GD algorithm has been used. The research employed both BGD and SGD algorithms to predict CO_2_ emissions in Italy with GDP growth as the input variable. More importantly, the research has optimized the prediction of CO_2_ emissions by using GDP growth as the input variable. According to the study findings, the BGD optimizer performed better than the SGD for prediction purposes. Additionally, the exploratory analysis result indicates a non-linear relationship between the two variables (Fig. 3). Therefore, the study suggests using more advanced machine learning methods like ANNs, which can perform better in learning non-linear patterns and complex mathematical mappings. For this reason, we use a MLP model in the next paragraph.

## ANN and MLP models

**Fig. 4:**
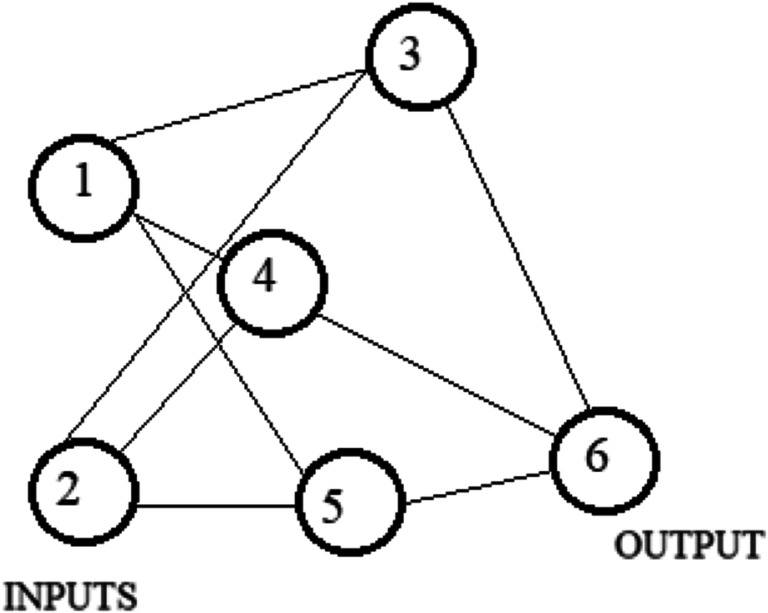
NN linear effect. Source: our elaborations in YeD

**Fig. 5:**
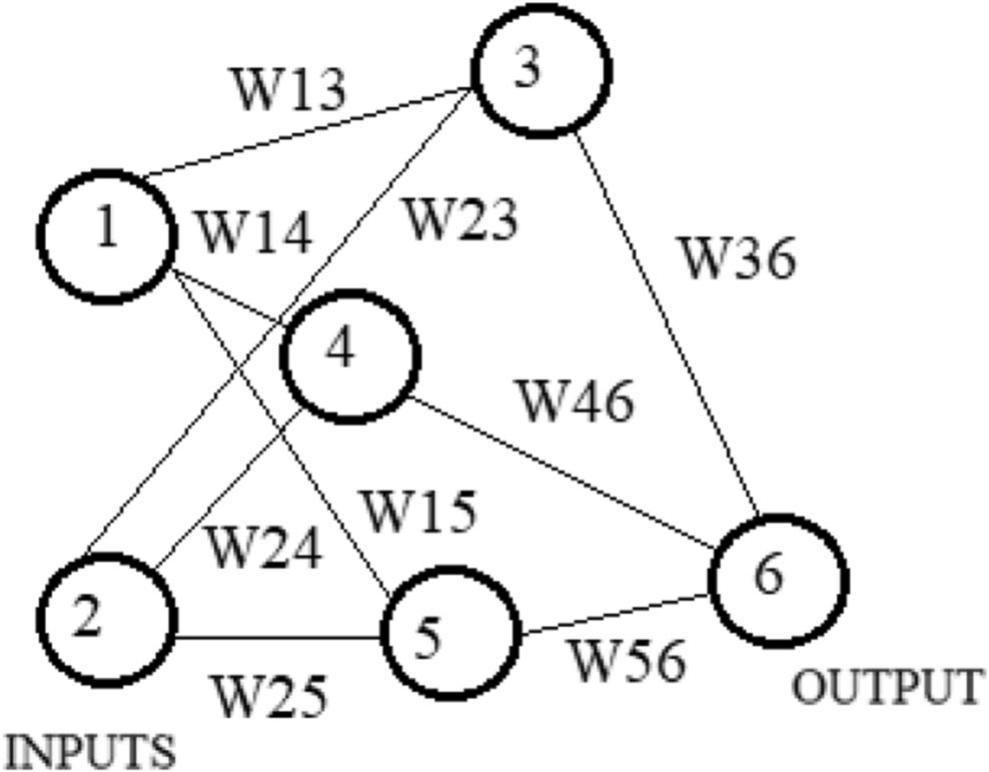
NN with the connection weights. Notes: W(x-y) represents the weight from node X to node Y. Source: our elaborations in YeD

ANNs are information processing systems. They try to simulate, within a computer system, the functioning of the biological nervous systems, which are made up of many nerve cells or neurons connected in a complex network. Each neuron is connected, on average, with tens of thousands of other neurons. So, there are hundreds of billions of connections. Intelligent behavior emerges from the numerous interactions between the interconnected units. Some of these units receive information from the environment, others emit responses in the environment, and still others—if any—communicate only with the units within the network: they are respectively defined as input (input), output (output), and hidden (hidden) units. Each unit performs an effortless operation consisting of becoming active if the total quantity of signal it receives exceeds a search activation threshold. If a unit becomes active, it emits a signal which is transmitted along the communication channels to the other units to which it is connected; each connection point acts as a filter that transforms the message received in an inhibitory or excitatory signal, increasing or decreasing its intensity at the same time according to one’s characteristics. Many neural networks carry out their learning process from examples through mechanisms that modify the weight system of the connections of the internal layers concerning the input patterns that are presented to the network. In this way, artificial neural networks’ learning process takes place in the same way as their biological counterparts. In Figs. 4 and 5, we show the effect known in the literature as linear and backpropagation effect.

Figure 4 shows a simple neural network consisting of an input layer consisting of two nodes, a single internal layer consisting of three nodes, and the output layer consisting of a single node. Excluding the input layer, each node contains an input set. These inputs are multiplied with Wxy connection weights (e.g., the weight from node 1 to node 3 is W13), and, adding the values obtained, the activation function associated with that node is applied to these values, and the output is transferred obtained at the node or nodes in the next layer. For example, the value transferred from node 4 to node 6 is activation function applied to ([W14 * value of node 1] + [W24 * value of node 2]).

The NN with the connection weights is shown in Fig. 5. Each node can be viewed as a predictor variable or as a combination of predictor variables. In addition, a neural network is somehow a mechanism that arises from a very complex generalization of a simple linear regression, which, in some cases, can be reduced to the latter. Now, we consider the functioning of the network training algorithm, which consists of identifying a reasonable estimate for the Wxy weights; it comprises the following two phases:

- *Feedforward*: in which the values of the node or output nodes are calculated on the basis of the input nodes and an initial set of weights. The values of the inputs nodes are combined in the internal nodes, and the values of these nodes are then combined to calculate the value or values of outputs.
- *Backpropagation*: this is the real phase of parameter estimation. In it, the error in the output is calculated by finding the difference between the output obtained from the network and the desired output (i.e., the actual values of the training set). Subsequently, the error obtained in the output is assigned to each node proportionally to their weights. This allows you to calculate an error for each output node and for each internal node so that the error in each of the output and internal nodes is used by the algorithm to adjust the weight in that node in order to reduce the total error. This learning process, consisting of a progressive decrease in the total network error, is repeated for each line of the training set. The algorithm’s step consisting of the use of all the lines of the training set is called epoch, and the algorithm will carry out several epochs on the training set repeatedly until the network error no longer decreases. However, the excessive number of nodes present in the internal layers of a generic neural network with the following high number of parameters almost always allows, and with a sufficiently high number of epochs, an almost complete adaptation of the network to the training set data.

Regarding the perceptron, it is a network formed by a number *m* of neurons. If we consider *d* as the number of inputs, the output of this type of network will, therefore, be given by:

1 $$ {y}_j=y\ \left({\sum}_{i=0}^d{w}_{ji}{x}_i\right) $$

where *x*_*i*_ are the inputs and *w*_*ji*_ are the weights of each input combined with each output. With this architecture, it is usually necessary to use the activation functions of the threshold function type. However, this type of network is quite limiting; therefore, other levels of neurons are introduced, and the activation function is replaced with the logistic function (otherwise, it would be not very easy to find a learning algorithm). In this way, being the logistic function differentiable, we can use the rules of differential calculus in the learning algorithm, generating the so-called multilayer perceptron. For example, let us consider a network made up of two levels of processing units, always with *d* inputs and *m* outputs for the first layer, but we also add *c* outputs to the second level. The second-level units are called hidden units, as their activation functions are not directly accessible from the outside. The final outputs of the network are, therefore, verifiable by the following expression:

2 $$ {z}_k=z\ \left(\ {\sum}_{j=0}^m{w}_{kj}^{\prime }{y}_j\right) $$

where *z*_*k*_ is the final output, w’*kj* are the weights for each processing unit, and *y*_*j*_ is the signal sent by the hidden units. The bias, as a coefficient respectively of the input *x*_0_ and *y*_0_, has been calculated by setting them equal to 1. Then, combining the above equations, we can write the final result:

3 $$ {z}_k=z\ \left(\ {\sum}_{j=0}^m{w}_{kj}^{\prime }y\left(\ {\sum}_{i=0}^d{w}_{ji}^{\prime }{x}_i\right)\right) $$

**Fig. 6:**
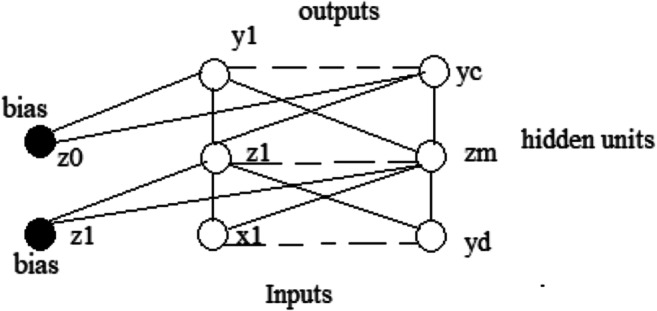
Multilayer perceptron. Source: our elaborations in YeD

Figure 6 shows the graphical representation of the previous equation.

## MLP algorithm^2^

The algorithms used to perform the analysis, with the same dataset of the GD model, are the following:

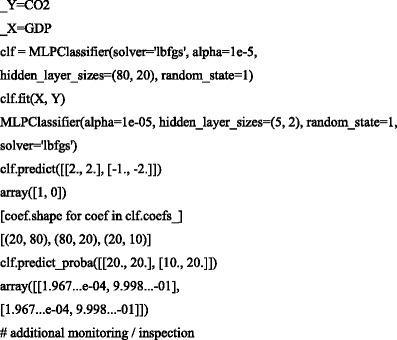



The previous commands represent a summary of the calculation used by us on the MLP function. In other words, we generated neural network training. The goal is, given a set of input vectors *x* and targets *t*, to make our network learn the relationship from which inputs and outputs are linked. Therefore, if we introduce (after training) an input to the network, the output will be very close to the target. To understand how the MLP algorithm programmed by us works, we first introduce the weight space, which is the size space of the network parameters. This space has dimension *n*, where *n* is the number of weights in the network. On the Cartesian axes, there are the weights (*w*_*1*_, *w*_*2*_, *...*, *w*_*n*_), and, therefore, each point of the plane corresponds to an exact function of the network. In fact, changing the weights also changes the network map. Once the weight space has been introduced, we can examine the error function, which is a function belonging to the weight space that measures how reliable the network is in solving the problem under consideration. The task of the learning algorithm is to minimize this function and then to find the point in the weight space where the function has the global minimum point.

To represent a generalized function of our model, we now consider an input vector (${x}_1^q,{x}_2^q,\dots, {x}_n^q\Big)$ and a target vector *t*^*q*^. We, therefore, take the sum of the squares of the residuals as errors. The residue is defined as *r*_*qk*_ = *y*_*k*_(*x*^*q*^, *w*) − *t*^*q*^; therefore, the error function will be expressed in this way:

4 $$ E=\frac{1}{2}{\sum}_{q=1}^n{\sum}_{k=1}^c\left\{{y}_k\left({x}^q,w\right)-{t}^q\Big\}{}^2\right. $$

By applying the derivative concerning the weights that go from the hidden level to the output, we can solve the previous expression:

5 $$ \frac{\partial {\mathrm{E}}^{\mathrm{q}}}{\partial {{\mathrm{w}}_{\mathrm{k}}^{\prime}}_{\mathrm{j}}}=\frac{\partial {\mathrm{E}}^{\mathrm{q}}}{\partial {a}_{\mathrm{k}}^{\prime }}\frac{\partial {a}_{\mathrm{k}}^{\prime }}{\partial {w}_{\mathrm{k}\mathrm{j}}^{\prime }} $$

where ${a}_{\mathrm{k}}^{\prime }$ is the activation of the final layer, therefore:

6 $$ {a}_k^{\prime }={\sum}_{j=0}^m{\mathrm{w}}_{\mathrm{k}\mathrm{j}}^{\prime }\ {z}_j\to {\delta}_k^{\prime }=\frac{\partial {\mathrm{E}}^{\mathrm{q}}}{\partial {a}_k^{\prime }}\to \frac{\partial {\mathrm{E}}^{\mathrm{q}}}{\partial {{\mathrm{w}}_{\mathrm{k}}^{\prime}}_{\mathrm{j}}}={\delta}_k^{\prime }{z}_j $$

combining the Eqs. (5) and (6), we have:

7 $$ {\delta}_k^{\prime }=z\left({a}_k^{\prime}\right)\ \left\{{y}_k-{t}_k\Big\}\right. $$

Since Eq. (7) is a difference between output and target values, it is therefore called “error.” As for the first level of processing units, let us start by writing the activation of the hidden units:

8 $$ {z}_j=g\left({a}_j\right),{a}_j={\sum}_{i=0}^d{w}_{ji}\ {x}_i\to \frac{\partial {\mathrm{E}}^{\mathrm{q}}}{\partial {{\mathrm{w}}_{\mathrm{k}}^{\prime}}_{\mathrm{j}}}={\delta}_k{x}_i\kern0.5em \mathrm{where}\ {\delta}_j={g}^{\prime}\left({a}_j\right){\sum}_{k=1}^c{w}_{ji}^{\prime }{\delta}_k^{\prime } $$

Substantially the error at the process unit *j* is given by the sum of the errors ${\delta}_k^{\prime }$ in the outputs multiplied by their weight. It is the backpropagation error.

**Fig. 7:**
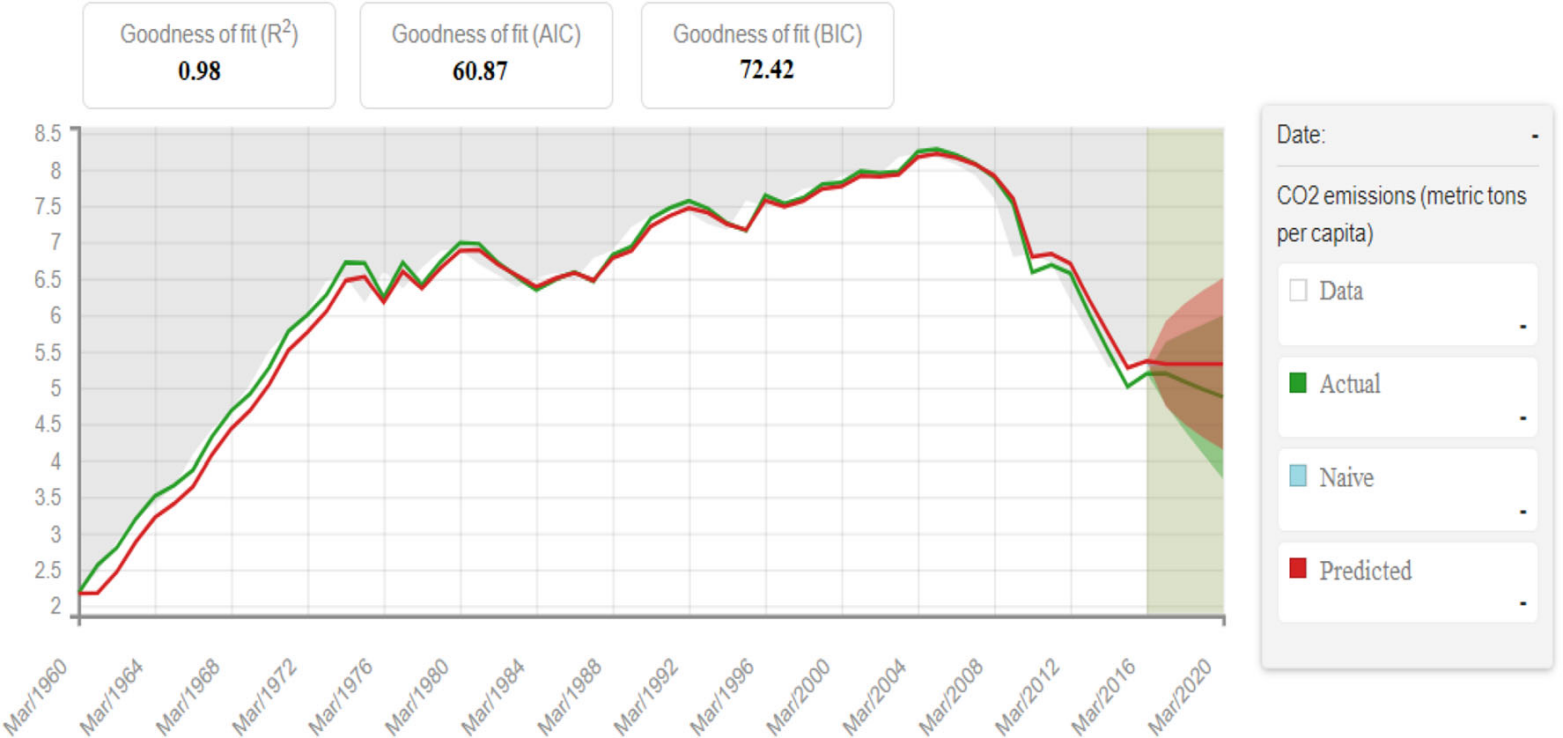
Predictions using MLP versus actual CO_2_ emissions. Source: our elaborations in BigML

**Fig. 8:**
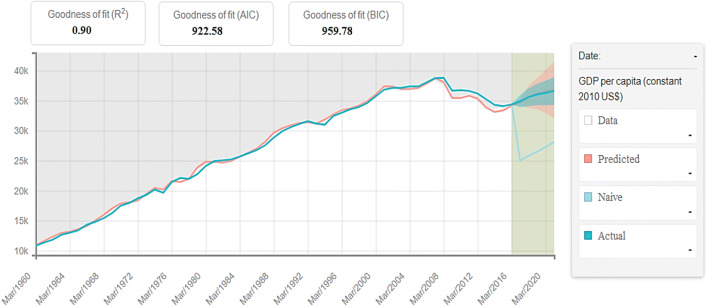
Predictions using MLP versus actual GDP per capita growth. Source: our elaborations in BigML

After training our program, we tried to predict CO_2_ emissions and GDP per capita growth in Italy. Figure 7 presents the predicted values of CO_2_ emission by the proposed MLP model against the actual values. Figure 8, on the other hand, analyzes the predicted values about GDP per capita proposed by our algorithm against the current values.

In Fig. 7, the predicted values were similar to the actual values only from March 1996 to 2008. Starting in 2008 to 2020, we can see a gap between the two lines. In particular, the predicted values by our algorithm (red line) are higher than the actual values (green line). This phenomenon is particularly evident after 2016 where, in the brown areas, the two lines diverge widely. The explanation for this phenomenon is as follows. From 2008 to 2014, in particular, the international financial crisis generated a phase of economic decline. Since 2011, Italy has also undergone the sovereign debt crisis in Europe. This event also influenced economic growth and the ability to invest in environmentally sustainable production. Therefore, the policymakers’ objective would have been to allow Italy a new phase of economic growth, with less concern for the environment. To verify what we are saying, let us analyze our algorithm’s result on the per capita GDP growth trend.

As we can see from Fig. 8, the levels of the predicted values are similar to the current values only until March 2008. However, we highlight how the values predicted in recent years, 2010–2017, are lower than the current ones. This result shows a per capita GDP decrease trend that is more significant than it actually is. The explanation for this result lies in the Italian economy’s cyclical trend that the algorithm is able to capture. The economic policies implemented in recent years are not suitable for a new economic growth phase after the previously mentioned financial and economic crises. Additionally, the negative trend of economic growth confirms the result obtained for CO_2_ emissions. The lower economic growth has pushed CO_2_ emissions, confirming the theory that sustainable production and renewable energy sources are not the main choices of policymakers in the phases of economic stagnation.

**Fig. 9:**
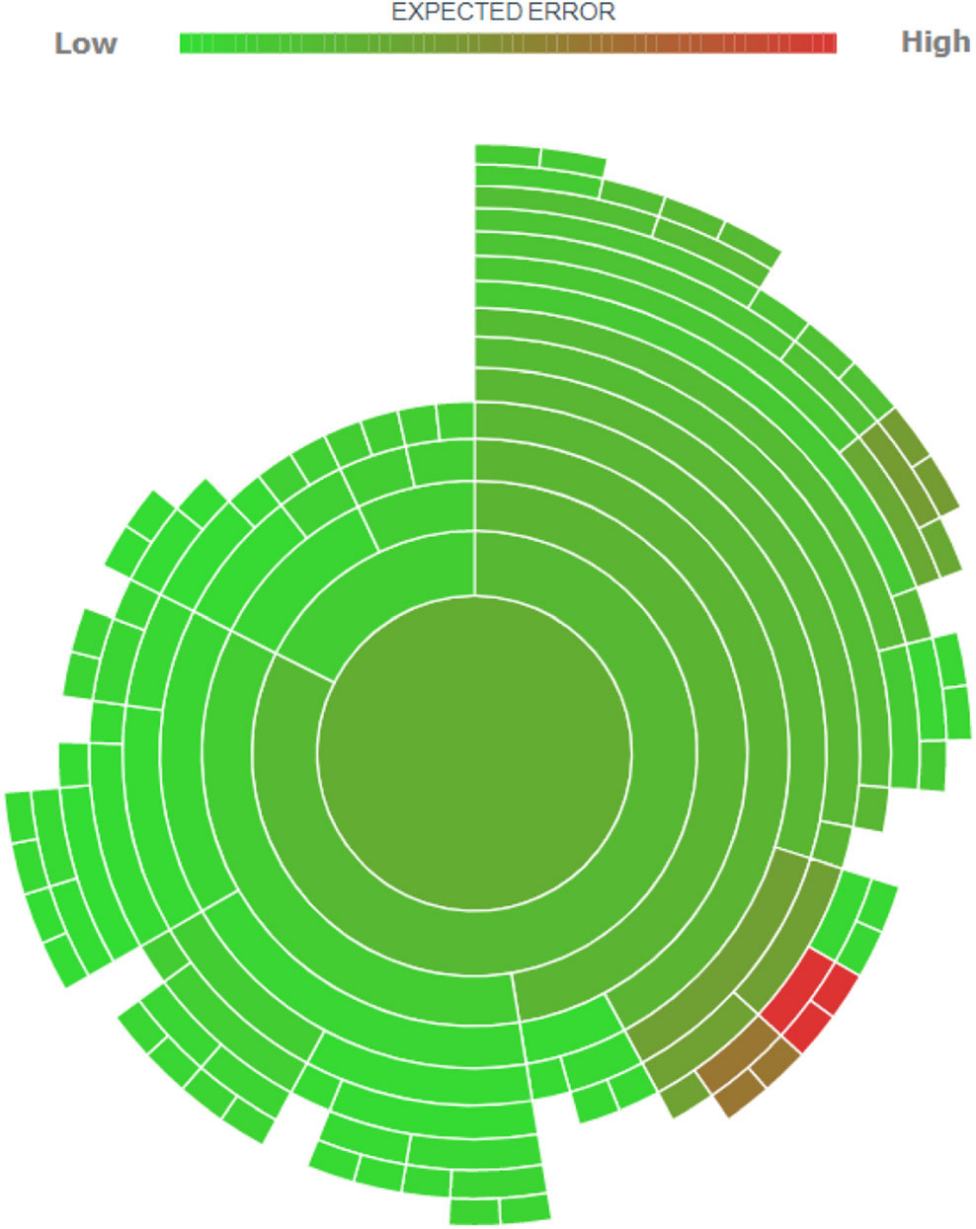
Sunburst ML test. Source: our elaborations in BigML

To verify the goodness of the algorithm’ results, we carry out the Sunburst ML test (Fig. 9). This test verifies the infinite combinations of prediction of the algorithm used concerning the set dataset. The test divides the combinations into numerous circular areas, representing the predictive phases from the beginning to the end of the elaboration. If the predictive analysis has a low calculation error, the algorithm is colored in a green cell. As the error increases, the cell becomes darker and darker until it becomes dark red. In this case, the predictive error is maximum.

As we can see from the figure, the prediction error of our algorithm is low. In particular, the cells external to the test’s circular flow have shallow errors (light green). Only six cells out of 120 total have a high prediction error. They, therefore, represent 5% of the predictive cells of our algorithm. The six cells with top predictive errors lie at the ends of the test. The central cells and the last main cell have a green color and underlines how a low error characterizes our algorithm.

Based on the Sunburst ML test results, we can say that the prediction analysis on CO_2_ emissions and per capita GDP are correct. Therefore, to verify the direct link between the predictive values ​​obtained, we have reset our algorithm. In particular, we have tried to ascertain the essence of a predictive correlation between the values predicted in Figs. 7 and 8. This verification represents a more precise explanation of the relationships between the two variables that are the subject of our study. A close graphic correlation would underline the social hypothesis that a phase of lower GDP growth has been crucial for the increase in CO_2_ in recent years.

**Fig. 10:**
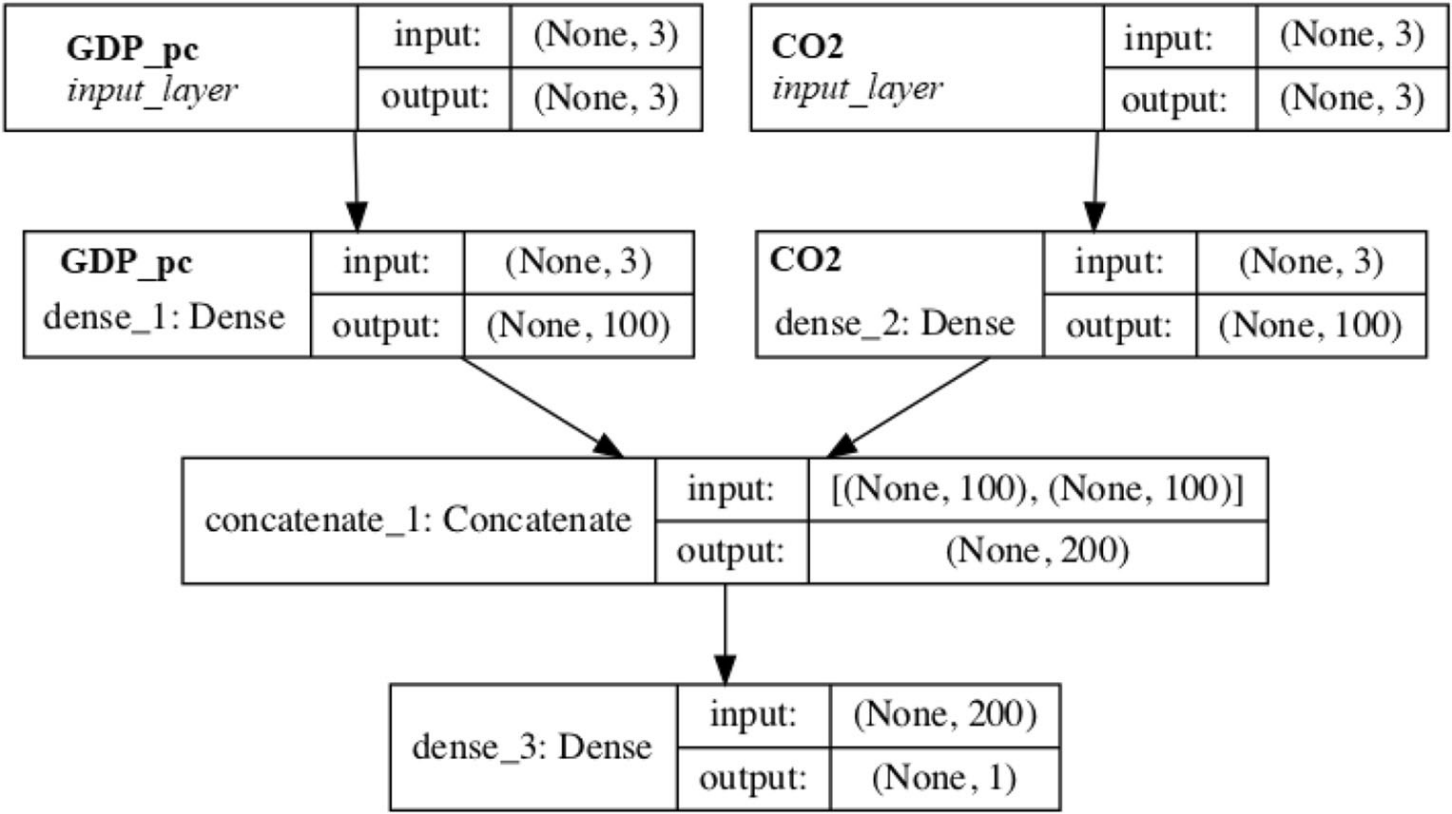
MLP NN process. Source: our elaborations in YeD

To this end, in our algorithm, we have divided the time series into five eras. Subsequently, we elaborated on a process of analysis on two inputs’ densities crossed with two outputs. In this way, we created a neural network on the infinite possibilities of predicting the correlation between per capita GDP per capita growth and CO_2_ emissions. The scheme of the new process is shown in Fig. 10.

Following the process depicted Fig. 10, we transcribe the new commands to process the predictive correlation. Therefore, we add the #bigml package to the program language.

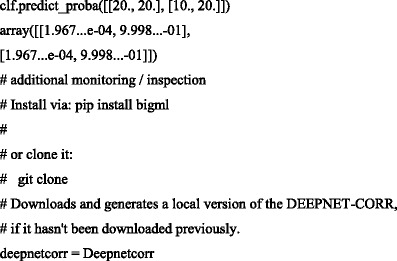



**Fig. 11:**
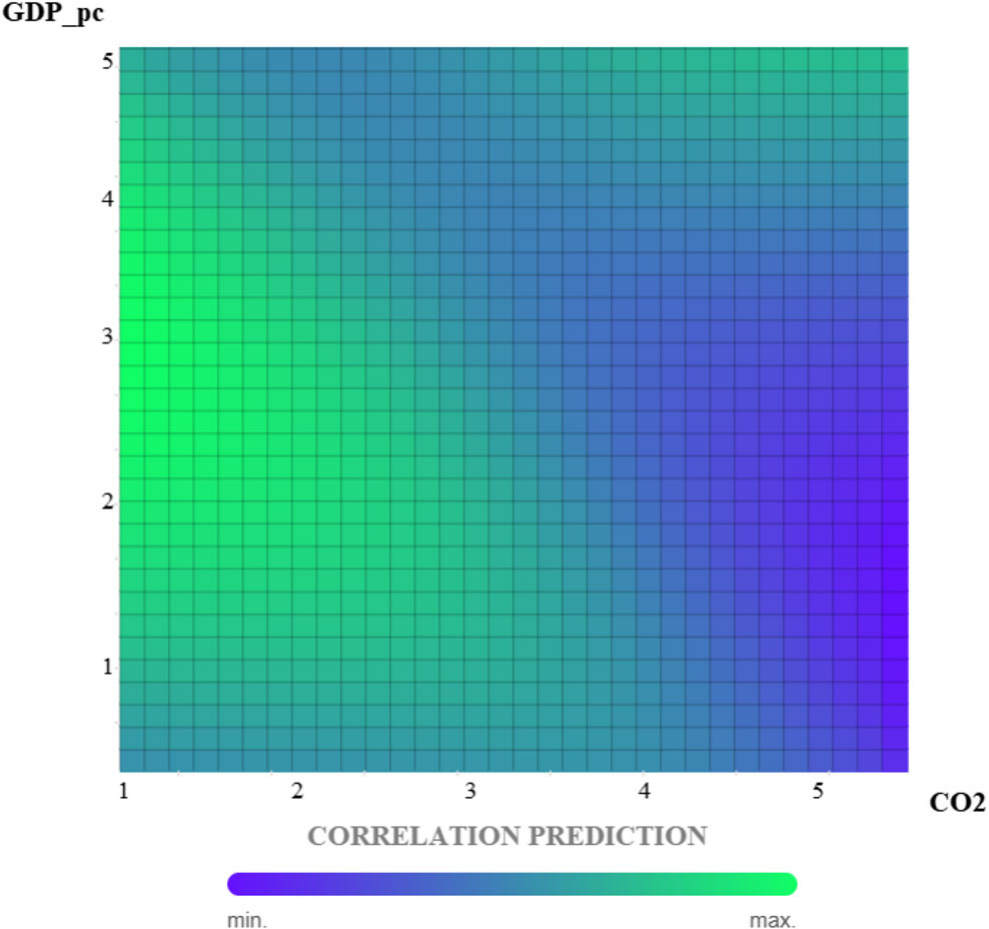
MLP correlation prediction, (In Appendix: Confusion Matrix). Source: our elaborations in BigML

As we can see from Fig. 11, the algorithm detects the correlation between per capita GDP and CO_2_ emissions predicted values. The values shown on the abscissa and ordinate axes (1–5) represent the algorithm’s 5 eras. The areas of the predictive correlation quadrant show a color trend ranging from light green to dark blue. When a correlation between our variables is in the green area, the greater is the correct degree of prediction of our algorithm. Therefore, looking at the graphical results, we notice that the maximum predictive correlation lies within the combination CO_2_-GDP and GDP-CO_2_: 2-1, 2-2, 2-3, 2-4, 3-2, 3-3. In the remaining combinations of ages 4 and 5, our predictive correlation has shallow values. However, the algorithm registers a new high predictive reaction as we reach the end of era number 5. This result confirms our hypothesis that the evolution of GDP (which has slowed down in recent years) has influenced CO_2_ emissions and CO_2_ emissions in Italy. While GDP slows down, at the same time national carbon dioxide emissions are increasing, driven by the methane and coal-fired thermal power plants.

## Concluding remarks and policy implications

In recent decades, gas emissions have created a hood in the upper layers of the atmosphere, creating the same overheating that occurs in greenhouses. The main culprit for this phenomenon is carbon dioxide. Regarding Italy, it is yet known that this economy will not reach its environmental target setting a 20% reduction of carbon emissions front. Nonetheless, GHG emissions have been experiencing a declining trend since 1990, corresponding to a 15.92% reduction. This might either find explanation in the recent EU crisis or can be traced back to the long-run deployment of low-carbon strategies across the territory and the novel emergence of a delinking dynamic among economic and environmental indicators.

Facing this issue, this paper presents the first empirical assessment of the relationship between economic growth and CO_2_ emissions using an innovative ML approach in Italy. To do so, three distinct models derived from AI are developed. Data span the largest available period (1960–2016). The results obtained in the prediction of the data with respect to the real values are highly interesting. Here, our novel BGD and SDG procedures verify to what extent the increase in CO_2_ emissions recorded is explained by a reduction in per capita GDP growth. This contradicts with a main strand of the economic theory and as our findings highlight that any negative shock in economic growth translates directly and significantly into rising environmental pollution and not vice versa. On the contrary, our MLP algorithms provide evidence of a robust correlation between the variables included in our study, which further confirms the robustness of our findings. Accordingly, these results imply that, in the face of a decrease in GDP, the capacity for environmentally sustainable investments in Italy may also decrease, inducing counter-intuitive effects on sustainability targets. Operating a break with the “de-growth” pattern, this study underlines here that reducing economic activity might not directly translate into lower emissions. In fact, a country with a high public debt such as Italy, in times of recession or low economic growth, relies on additional resources to pay interest rates on public debt. This divestment effect adversely impacts pollution trends since low-carbon investments fall as economic activity slows down. Therefore, we claim here that public budget choices should be reconsidered by directing the decisions toward a qualitative and non-quantitative public budget. In doing so, funding and state aid to more environmentally sustainable production could be included and further delink environmental benefits (losses) from economic performance (recession).

## Supplementary Information

ESM 1 (DOCX 34 kb)

## Data Availability

If requested, we are available to disseminate the data of the paper.

## Footnotes

1 https://databank.worldbank.org/source/world-development-indicators

2 We only publish the main commands. The complete algorithm programmed by us remains available only for further checks.
