# Supplementary material for: Revisiting the dynamic interactions between economic growth and environmental pollution in Italy: evidence from a gradient descent algorithm
Source: Environ Sci Pollut Res Int. 2021 May 18;28(37):52188–201. doi: 10.1007/s11356-021-14264-z (PMC8458215; doi:10.1007/s11356-021-14264-z)
Supplement: Supplementary file 1 — (DOCX 34 kb) [file 11356_2021_14264_MOESM1_ESM.docx]

**Appendix**

*A) Confusion Matrix*

|  | **Predicted Positive** | |  | | **Predicted Negative** | | |  |
| --- | --- | --- | --- | --- | --- | --- | --- | --- |
| **Actual Positive** | | 6078 | |  | | 338 |  | |
| **Actual Negative** | | 309 | |  | | 5582 |  | |

*B) MPL Data distribution*

- Data distribution:
- **10867.70366**: **1.75**% (**1** instance)
- **11681.30547**: **1.75**% (**1** instance)
- **12322.31451**: **1.75**% (**1** instance)
- **13192.8532**: **5.26**% (**3** instances)
- **14184.53188**: **1.75**% (**1** instance)
- **15093.14726**: **1.75**% (**1** instance)
- **15979.65754**: **1.75**% (**1** instance)
- **16858.40759**: **1.75**% (**1** instance)
- **17771.66828**: **3.51**% (**2** instances)
- **18445.54557**: **1.75**% (**1** instance)
- **19823.65809**: **3.51**% (**2** instances)
- **20570.81838**: **1.75**% (**1** instance)
- **21567.59973**: **3.51**% (**2** instances)
- **22420.57388**: **1.75**% (**1** instance)
- **23688.05915**: **1.75**% (**1** instance)
- **24694.49936**: **7.02**% (**4** instances)
- **25790.74798**: **1.75**% (**1** instance)
- **26504.73426**: **1.75**% (**1** instance)
- **27261.27663**: **1.75**% (**1** instance)
- **28128.57514**: **1.75**% (**1** instance)
- **29294.23513**: **1.75**% (**1** instance)
- **30264.12683**: **1.75**% (**1** instance)
- **30839.27833**: **1.75**% (**1** instance)
- **31355.80738**: **5.26**% (**3** instances)
- **31909.23671**: **1.75**% (**1** instance)
- **33036.36752**: **3.51**% (**2** instances)
- **33824.4092**: **7.02**% (**4** instances)
- **34384.81651**: **3.51**% (**2** instances)
- **35050.31983**: **5.26**% (**3** instances)
- **36008.09504**: **5.26**% (**3** instances)
- **36935.68008**: **8.77**% (**5** instances)
- **37898.10044**: **5.26**% (**3** instances)
- Predicted distribution:
- **10867.70366**: **1.75**% (**1** instance)
- **11681.30547**: **1.75**% (**1** instance)
- **12322.31451**: **1.75**% (**1** instance)
- **12918.96745**: **1.75**% (**1** instance)
- **13171.60209**: **1.75**% (**1** instance)
- **13487.99005**: **1.75**% (**1** instance)
- **14184.53188**: **1.75**% (**1** instance)
- **15093.14726**: **1.75**% (**1** instance)
- **15979.65754**: **1.75**% (**1** instance)
- **16858.40759**: **1.75**% (**1** instance)
- **17653.01526**: **1.75**% (**1** instance)
- **17890.32129**: **1.75**% (**1** instance)
- **18445.54557**: **1.75**% (**1** instance)
- **19626.3936**: **1.75**% (**1** instance)
- **20020.92257**: **1.75**% (**1** instance)
- **20570.81838**: **1.75**% (**1** instance)
- **21340.7658**: **1.75**% (**1** instance)
- **21794.43366**: **1.75**% (**1** instance)
- **22420.57388**: **1.75**% (**1** instance)
- **23688.05915**: **1.75**% (**1** instance)
- **24450.14431**: **1.75**% (**1** instance)
- **24626.97568**: **1.75**% (**1** instance)
- **24710.51652**: **1.75**% (**1** instance)
- **24990.36094**: **1.75**% (**1** instance)
- **25790.74798**: **1.75**% (**1** instance)
- **26504.73426**: **1.75**% (**1** instance)
- **27261.27663**: **1.75**% (**1** instance)
- **28128.57514**: **1.75**% (**1** instance)
- **29294.23513**: **1.75**% (**1** instance)
- **30264.12683**: **1.75**% (**1** instance)
- **30839.27833**: **1.75**% (**1** instance)
- **31243.67902**: **1.75**% (**1** instance)
- **31292.05309**: **1.75**% (**1** instance)
- **31531.69002**: **1.75**% (**1** instance)
- **31909.23671**: **1.75**% (**1** instance)
- **32829.88275**: **1.75**% (**1** instance)
- **33242.8523**: **1.75**% (**1** instance)
- **33615.97178**: **1.75**% (**1** instance)
- **33835.07313**: **1.75**% (**1** instance)
- **33887.29817**: **1.75**% (**1** instance)
- **33959.29372**: **1.75**% (**1** instance)
- **34371.98202**: **1.75**% (**1** instance)
- **34397.65099**: **1.75**% (**1** instance)
- **34885.2969**: **1.75**% (**1** instance)
- **34902.26213**: **1.75**% (**1** instance)
- **35363.40046**: **1.75**% (**1** instance)
- **35849.3732**: **1.75**% (**1** instance)
- **35994.13358**: **1.75**% (**1** instance)
- **36180.77833**: **1.75**% (**1** instance)
- **36729.97554**: **1.75**% (**1** instance)
- **36801.29381**: **1.75**% (**1** instance)
- **36837.86399**: **1.75**% (**1** instance)
- **37070.33052**: **1.75**% (**1** instance)
- **37238.93653**: **1.75**% (**1** instance)
- **37585.33735**: **1.75**% (**1** instance)
- **37872.16836**: **1.75**% (**1** instance)
- **38236.79561**: **1.75**% (**1** instance)

*2) Sunburst ML test*

- def predict_gdp_per_capita_constant_2010_us_(co2_emissions_metric_tons_per_capita_=None):
- if (co2_emissions_metric_tons_per_capita_ is None):
- return **27097.2291**
- if (co2_emissions_metric_tons_per_capita_ > **5.15709**):
- if (co2_emissions_metric_tons_per_capita_ > **7.02592**):
- if (co2_emissions_metric_tons_per_capita_ > **7.5874**):
- if (co2_emissions_metric_tons_per_capita_ > **7.91501**):
- if (co2_emissions_metric_tons_per_capita_ > **7.92484**):
- if (co2_emissions_metric_tons_per_capita_ > **8.16892**):
- if (co2_emissions_metric_tons_per_capita_ > **8.19412**):
- return **37070.33052**
- if (co2_emissions_metric_tons_per_capita_ <= **8.19412**):
- return **36729.97554**
- if (co2_emissions_metric_tons_per_capita_ <= **8.16892**):
- if (co2_emissions_metric_tons_per_capita_ > **8.00223**):
- if (co2_emissions_metric_tons_per_capita_ > **8.11912**):
- return **37238.93653**
- if (co2_emissions_metric_tons_per_capita_ <= **8.11912**):
- return **37872.16836**
- if (co2_emissions_metric_tons_per_capita_ <= **8.00223**):
- return **36837.86399**
- if (co2_emissions_metric_tons_per_capita_ <= **7.92484**):
- return **38236.79561**
- if (co2_emissions_metric_tons_per_capita_ <= **7.91501**):
- if (co2_emissions_metric_tons_per_capita_ > **7.66821**):
- if (co2_emissions_metric_tons_per_capita_ > **7.83509**):
- if (co2_emissions_metric_tons_per_capita_ > **7.90855**):
- return **36180.77833**
- if (co2_emissions_metric_tons_per_capita_ <= **7.90855**):
- return **36801.29381**
- if (co2_emissions_metric_tons_per_capita_ <= **7.83509**):
- if (co2_emissions_metric_tons_per_capita_ > **7.7502**):
- return **34902.26213**
- if (co2_emissions_metric_tons_per_capita_ <= **7.7502**):
- return **34371.98202**
- if (co2_emissions_metric_tons_per_capita_ <= **7.66821**):
- return **37585.33735**
- if (co2_emissions_metric_tons_per_capita_ <= **7.5874**):
- if (co2_emissions_metric_tons_per_capita_ > **7.47872**):
- if (co2_emissions_metric_tons_per_capita_ > **7.57**):
- return **32829.88275**
- if (co2_emissions_metric_tons_per_capita_ <= **7.57**):
- if (co2_emissions_metric_tons_per_capita_ > **7.52802**):
- return **33835.07313**
- if (co2_emissions_metric_tons_per_capita_ <= **7.52802**):
- return **33242.8523**
- if (co2_emissions_metric_tons_per_capita_ <= **7.47872**):
- if (co2_emissions_metric_tons_per_capita_ > **7.19102**):
- if (co2_emissions_metric_tons_per_capita_ > **7.23169**):
- if (co2_emissions_metric_tons_per_capita_ > **7.38294**):
- if (co2_emissions_metric_tons_per_capita_ > **7.43627**):
- return **31292.05309**
- if (co2_emissions_metric_tons_per_capita_ <= **7.43627**):
- return **31531.69002**
- if (co2_emissions_metric_tons_per_capita_ <= **7.38294**):
- if (co2_emissions_metric_tons_per_capita_ > **7.30486**):
- return **30839.27833**
- if (co2_emissions_metric_tons_per_capita_ <= **7.30486**):
- return **31243.67902**
- if (co2_emissions_metric_tons_per_capita_ <= **7.23169**):
- return **30264.12683**
- if (co2_emissions_metric_tons_per_capita_ <= **7.19102**):
- return **31909.23671**
- if (co2_emissions_metric_tons_per_capita_ <= **7.02592**):
- if (co2_emissions_metric_tons_per_capita_ > **5.43904**):
- if (co2_emissions_metric_tons_per_capita_ > **6.69649**):
- if (co2_emissions_metric_tons_per_capita_ > **6.85919**):
- if (co2_emissions_metric_tons_per_capita_ > **6.88151**):
- if (co2_emissions_metric_tons_per_capita_ > **6.88775**):
- return **24450.14431**
- if (co2_emissions_metric_tons_per_capita_ <= **6.88775**):
- return **29294.23513**
- if (co2_emissions_metric_tons_per_capita_ <= **6.88151**):
- return **23688.05915**
- if (co2_emissions_metric_tons_per_capita_ <= **6.85919**):
- if (co2_emissions_metric_tons_per_capita_ > **6.78703**):
- if (co2_emissions_metric_tons_per_capita_ > **6.81701**):
- return **35849.3732**
- if (co2_emissions_metric_tons_per_capita_ <= **6.81701**):
- return **35363.40046**
- if (co2_emissions_metric_tons_per_capita_ <= **6.78703**):
- if (co2_emissions_metric_tons_per_capita_ > **6.74048**):
- return **28128.57514**
- if (co2_emissions_metric_tons_per_capita_ <= **6.74048**):
- return **35994.13358**
- if (co2_emissions_metric_tons_per_capita_ <= **6.69649**):
- if (co2_emissions_metric_tons_per_capita_ > **5.62317**):
- if (co2_emissions_metric_tons_per_capita_ > **5.74763**):
- if (co2_emissions_metric_tons_per_capita_ > **6.18987**):
- if (co2_emissions_metric_tons_per_capita_ > **6.28538**):
- if (co2_emissions_metric_tons_per_capita_ > **6.47317**):
- if (co2_emissions_metric_tons_per_capita_ > **6.50988**):
- if (co2_emissions_metric_tons_per_capita_ > **6.52975**):
- if (co2_emissions_metric_tons_per_capita_ > **6.58397**):
- if (co2_emissions_metric_tons_per_capita_ > **6.66846**):
- return **24626.97568**
- if (co2_emissions_metric_tons_per_capita_ <= **6.66846**):
- if (co2_emissions_metric_tons_per_capita_ > **6.6194**):
- return **22420.57388**
- if (co2_emissions_metric_tons_per_capita_ <= **6.6194**):
- return **21340.7658**
- if (co2_emissions_metric_tons_per_capita_ <= **6.58397**):
- if (co2_emissions_metric_tons_per_capita_ > **6.55681**):
- return **26504.73426**
- if (co2_emissions_metric_tons_per_capita_ <= **6.55681**):
- return **24710.51652**
- if (co2_emissions_metric_tons_per_capita_ <= **6.52975**):
- return **20570.81838**
- if (co2_emissions_metric_tons_per_capita_ <= **6.50988**):
- if (co2_emissions_metric_tons_per_capita_ > **6.48657**):
- return **25790.74798**
- if (co2_emissions_metric_tons_per_capita_ <= **6.48657**):
- return **27261.27663**
- if (co2_emissions_metric_tons_per_capita_ <= **6.47317**):
- if (co2_emissions_metric_tons_per_capita_ > **6.43001**):
- return **19626.3936**
- if (co2_emissions_metric_tons_per_capita_ <= **6.43001**):
- if (co2_emissions_metric_tons_per_capita_ > **6.37697**):
- return **24990.36094**
- if (co2_emissions_metric_tons_per_capita_ <= **6.37697**):
- return **21794.43366**
- if (co2_emissions_metric_tons_per_capita_ <= **6.28538**):
- return **34885.2969**
- if (co2_emissions_metric_tons_per_capita_ <= **6.18987**):
- if (co2_emissions_metric_tons_per_capita_ > **6.11182**):
- return **20020.92257**
- if (co2_emissions_metric_tons_per_capita_ <= **6.11182**):
- if (co2_emissions_metric_tons_per_capita_ > **5.90582**):
- return **18445.54557**
- if (co2_emissions_metric_tons_per_capita_ <= **5.90582**):
- return **17890.32129**
- if (co2_emissions_metric_tons_per_capita_ <= **5.74763**):
- return **33887.29817**
- if (co2_emissions_metric_tons_per_capita_ <= **5.62317**):
- return **17653.01526**
- if (co2_emissions_metric_tons_per_capita_ <= **5.43904**):
- if (co2_emissions_metric_tons_per_capita_ > **5.29867**):
- if (co2_emissions_metric_tons_per_capita_ > **5.34557**):
- return **33959.29372**
- if (co2_emissions_metric_tons_per_capita_ <= **5.34557**):
- return **34397.65099**
- if (co2_emissions_metric_tons_per_capita_ <= **5.29867**):
- return **33615.97178**
- if (co2_emissions_metric_tons_per_capita_ <= **5.15709**):
- if (co2_emissions_metric_tons_per_capita_ > **3.86047**):
- if (co2_emissions_metric_tons_per_capita_ > **4.55905**):
- if (co2_emissions_metric_tons_per_capita_ > **4.86496**):
- return **16858.40759**
- if (co2_emissions_metric_tons_per_capita_ <= **4.86496**):
- return **15979.65754**
- if (co2_emissions_metric_tons_per_capita_ <= **4.55905**):
- if (co2_emissions_metric_tons_per_capita_ > **4.25546**):
- return **15093.14726**
- if (co2_emissions_metric_tons_per_capita_ <= **4.25546**):
- return **14184.53188**
- if (co2_emissions_metric_tons_per_capita_ <= **3.86047**):
- if (co2_emissions_metric_tons_per_capita_ > **2.67153**):
- if (co2_emissions_metric_tons_per_capita_ > **3.0468**):
- if (co2_emissions_metric_tons_per_capita_ > **3.52328**):
- return **13487.99005**
- if (co2_emissions_metric_tons_per_capita_ <= **3.52328**):
- if (co2_emissions_metric_tons_per_capita_ > **3.31008**):
- return **13171.60209**
- if (co2_emissions_metric_tons_per_capita_ <= **3.31008**):
- return **12918.96745**
- if (co2_emissions_metric_tons_per_capita_ <= **3.0468**):
- return **12322.31451**
- if (co2_emissions_metric_tons_per_capita_ <= **2.67153**):
- if (co2_emissions_metric_tons_per_capita_ > **2.3215**):
- return **11681.30547**
- if (co2_emissions_metric_tons_per_capita_ <= **2.3215**):
- return **10867.70366**
